# Knowledge landscape of tumor-associated neutrophil: a bibliometric and visual analysis from 2000-2024

**DOI:** 10.3389/fimmu.2024.1448818

**Published:** 2024-10-04

**Authors:** Chaoyue Xiao, Xiang Feng, Wufuer Aini, Zengyi Zhao, Gouping Ding, Yawen Gao

**Affiliations:** ^1^ Department of Oncology, The Second Xiangya Hospital, Central South University, Changsha, China; ^2^ State Key Laboratory of Pathogenesis, Prevention and Treatment of High Incidence Diseases in Central Asia, Department of Endocrinology, The First Affiliated Hospital of Xinjiang Medical University, Urumqi, China

**Keywords:** tumor-associated neutrophil, cancer, immunology, bibliometrics, visualization, VOSviewer, CiteSpace, R-bibliometrix

## Abstract

**Background:**

Neutrophils have long been consistently adjudged to hold a dominant position in acute inflammation, which once led people to undervalue their role in chronic malignancy. It is now acknowledged that neutrophils also infiltrate into the tumor microenvironment in substantial quantities and form a highly abundant immune population within the tumor, known as tumor-associated neutrophils (TANs). There has been a surge of interest in researching the eminent heterogeneity and plasticity of TANs in recent years, and scholars increasingly cotton on to the multifaceted functions of TANs so that strenuous endeavors have been devoted to enunciating their potential as therapeutic targets. Yet it remains much left to translate TAN-targeted immunotherapies into clinical practice. Therefore, there is great significance to comprehensively appraise the research status, focal point, and evolution trend of TAN by using bibliometric analysis.

**Methods:**

Publications related to TAN research from 2000 to 2024 are extracted from the Web of Science Core Collection. Bibliometric analysis and visualization were performed by tools encompassing Microsoft Excel, VOSviewer, CiteSpace, R-bibliometrix, and so on.

**Results:**

The bibliometric analysis included a total of 788 publications authored by 5291 scholars affiliated with 1000 institutions across 58 countries/regions, with relevant articles published in 324 journals. Despite China’s maximum quantity of publications and top 10 institutions, the United States is the leading country with the most high-quality publications and is also the global cooperation center. FRONTIERS IN IMMUNOLOGY published the most papers, whereas CANCER RESEARCH is the highest co-cited journal. Israeli professor Fridlender, Zvi G. is the founder, pioneer, and cultivator with the highest citation counts and H-index in the TAN area. Our analysis prefigures the future trajectories: TAN heterogeneity, neutrophil extracellular trap, the crosstalk between TANs and immunocytes, and immunotherapy will likely be the focus of future research.

**Conclusion:**

A comprehensive bibliometric and visual analysis is first performed to map the current landscape and intellectual structure of TAN, which proffers fresh perspectives for further research. The accurate identification of distinct TAN subpopulations and the precise targeting of key pro-tumor/anti-tumor subpopulations hold immense potential to develop into a TAN-targeted immunotherapy.

## 1 Introduction

Comprehending the characteristics of immune cells in cancer is a progressive undertaking, with earlier studies mostly centered around adaptive immune cells. However, solely targeting the activating of T cells to boost the cytotoxic capacity of immune system has already demonstrated the miserable efficacy in addressing the underlying challenge of immunosuppression in the context of tumor (1, 2). Along with the deepening in perceptions of tumor microenvironment (TME), the attention of investigation has switched from adaptive immunity to innate immunity. As a fundamental component of the innate immune system, neutrophil is the predominant common population of polymorphonuclear leukocytes and accounts for 50-70% of circulating leukocytes in humans (3, 4). Neutrophil originates from granulocyte monocyte progenitor in bone marrow and is dispatched into circulation as mature cell distinguished by a segmented nucleus. As the front-line immunocyte defending against microbial infections, neutrophil holds a dominant position in the realm of inflammatory disorders, especially acute inflammation (5). Neutrophils possess two specific characteristics: an ephemeral lifespan and an inability to proliferate, which differentiates them from other immune cells. For instance, the half-life of circulating neutrophil is merely about 7 hours in humans. These peculiarities once led people to undervalue their role in tumors, which are chronic malignant diseases (6).

Nevertheless, as our perception of neutrophils has grown more profound, research has substantiated that their lifespan can extend up to 5.4 days through labeling with ^2^H_2_O *in vivo*. Neutrophils not only constitute the predominant population of circulating cells within the human bloodstream but also manifest a salient propensity for infiltration into the sophisticated milieu of the TME in substantial quantities, which could be actuated by external stimuli from TME, culminating in forming a highly abundant immune population within the TME, known as tumor-associated neutrophils (TANs) (7–10). TANs engage in TME with their phenotypic and functional plasticity. Specifically, the pro-tumor TANs are involved in tumor proliferation, angiogenesis, and immunosuppression within TME, thereby engaging in all stages of cancer progression. Whereas the anti-tumor TANs directly kill tumor cells with cytotoxicity or activate innate or adaptive immunities, thereby impeding cancer progression. Thus, there has been a surge of interest among scholars in researching the eminent heterogeneity and plasticity of TANs in recent years, and scholars increasingly cotton on to the multifaceted functions of TANs within the TME so that strenuous endeavors have been devoted to enunciating their potential as therapeutic targets (11–14). Correspondingly, the incipient boom of emerging TAN realm in recent years has achieved a series of scientific research outputs, which opens up a new perspective for perceiving the tumor immune microenvironment. Yet it remains much left to translate TAN-targeted immunotherapies into clinical practice. Therefore, there is great significance to comprehensively appraise the research status, focal point, and evolution trend of TAN.

Bibliometrics is an emerging interdisciplinary science that delivers a comprehensive and objective assessment of knowledge carriers by mathematics and statistics, in which the bibliographic analysis facilitates scholars to digest the progression of specific topics and evinces the evolution trend of this field. Nevertheless, bibliometric analysis on the TAN remains a void. Hence, this study aimed to map the current landscape and intellectual structure of TAN in the hope of proffering fresh clues and ideas for future research in the field of TAN.

## 2 Materials and methods

### 2.1 Data source and search strategy

**Figure 1:**
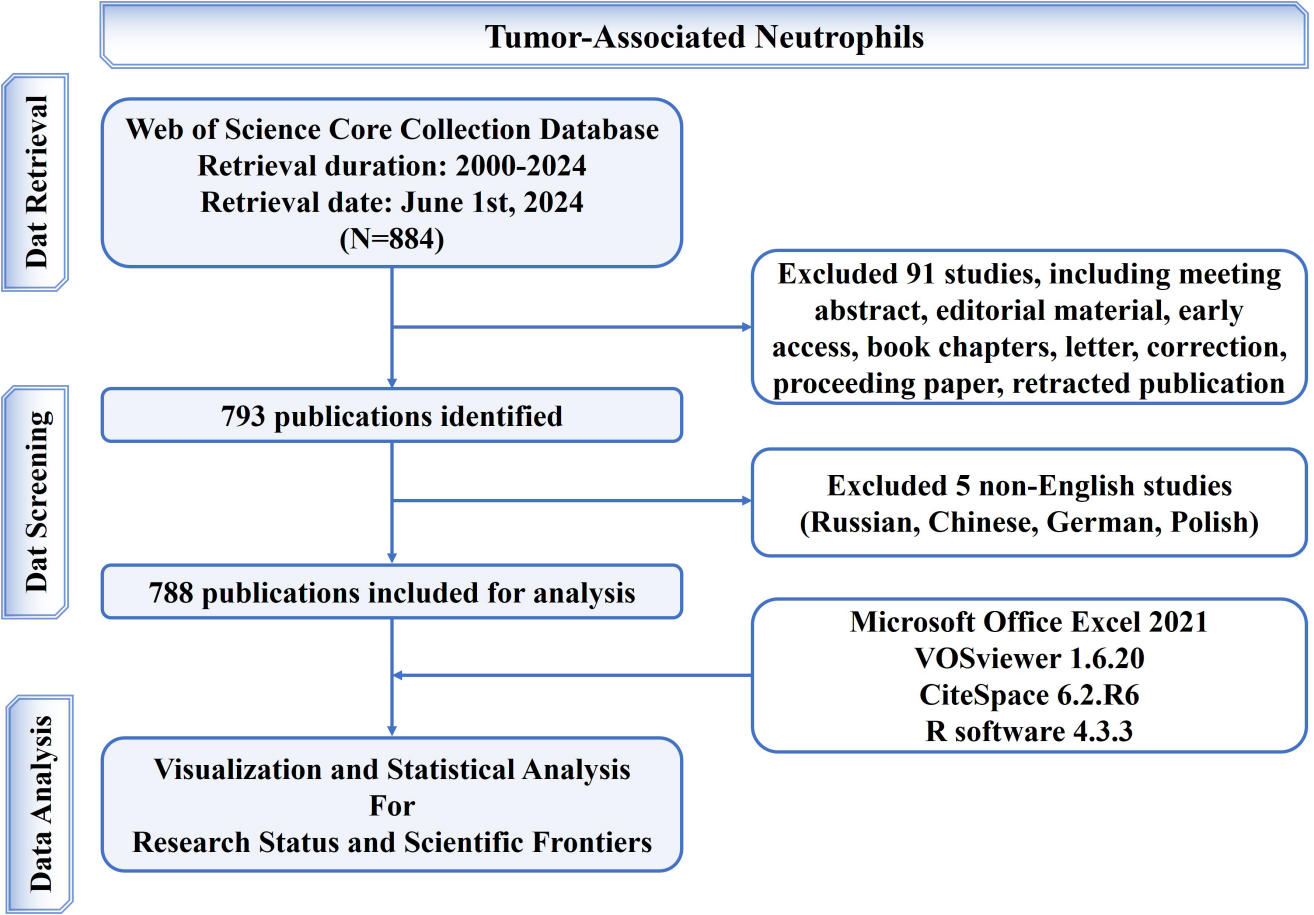
Flowchart of the screening process.

Scientific output data was extracted from the Web of Science Core Collection (WoSCC) database (https://www.webofscience.com/wos/woscc/basic-search). Two reviewers independently searched original articles and reviews from inception to 2024 and downloaded information of these publications in plain text format in a single day (2024.06.01). The search formula was set as follows: TS = (“tumor associated neutrophil*” OR “tumor-associated neutrophil*” OR “tumour associated neutrophil*” OR “tumour-associated neutrophil*” OR “cancer associated neutrophil*” OR “cancer-associated neutrophil*” OR “tumor related neutrophil*” OR “tumor-related neutrophil*” OR “tumour related neutrophil*” OR “tumour-related neutrophil*” OR “cancer related neutrophil*” OR “cancer-related neutrophil*”) OR “tumor infiltrating neutrophil*” OR “tumor-infiltrating neutrophil*” OR “tumour infiltrating neutrophil*” OR “tumour-infiltrating neutrophil*” OR “cancer infiltrating neutrophil*” OR “cancer-infiltrating neutrophil*”). Among the various forms of relevant publications, the English articles and reviews were the ones analyzed. Finally, 788 publications were accessed and studied in total. The study flowchart for accessing and retrieving articles and reviews has been elaborated in **Figure 1**.

### 2.2 Visualization and statistical analysis

Microsoft Office Excel 2021 (Microsoft, Redmond, WA, USA) was the prime software used to construct a polynomial regression model for the number of publications. VOSviewer (Version 1.6.20) is a generally used bibliometric visualization software, which was utilized to conduct a co-authorship analysis of country/author/institution, co-citation analysis of journal, and keyword co-occurrence analysis. CiteSpace (Version 6.2.R6) is another favored visualization tool, which was chiefly applied to provide a visual representation of the co-citation network identifying key references and the references with the strongest citation bursts in this study. Additionally, a dual-map overlay of journals was also created by CiteSpace. R software (Version 4.3.3) is the language and environment, which is extensively utilized for statistical computing and graphics. In this study, the bibliometrix package 4.1.2 in R and two online platforms (https://bibliometric.com/ and https://flourish.studio/examples/) were used to perform the collaboration analysis among countries/regions, authors’ production over time, document citation analysis, keyword heatmaps, trend themes, and so on. The appropriate parameters are set to ensure that the clustering and grouping effects are clearly visible in the use of the aforementioned tools. Nodes with more significant connections will be classified as a cluster, and different clusters will be colored differently.

## 3 Results

### 3.1 Research profile

**Figure 2:**
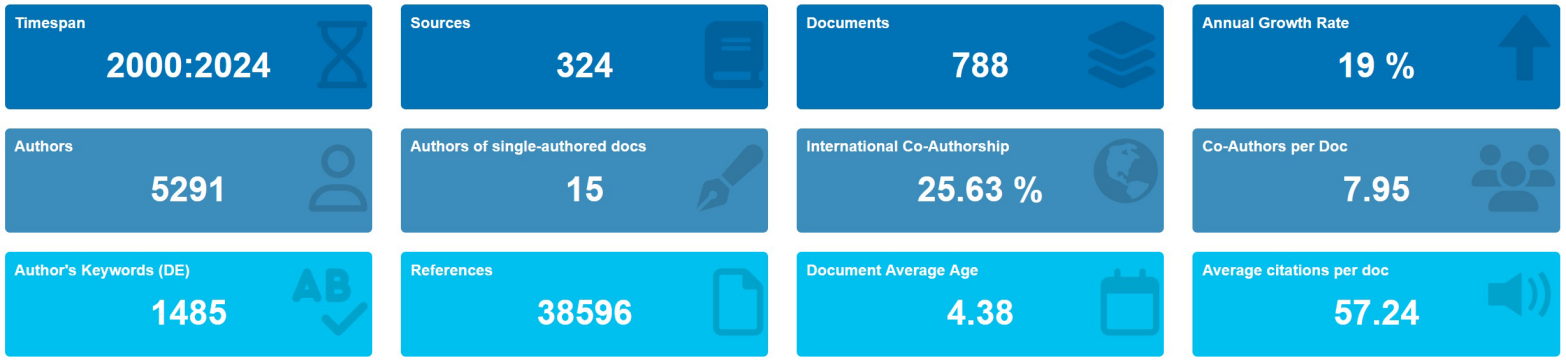
Basic information of 788 relevant publications included. The period of included articles, the number of journal categories, the total number of articles, the annual growth rate, the total number of authors, the number of articles published by a single author, the proportion of international co-authors, the number of co-authors of an article, the keywords given by the author, the number of references cited, the average life span of each article, the average number of citations per article.

According to the search strategy (**Figure 1**), 788 eligible publications from 2000 to 2024 are retrieved. The current status of TAN research is described using R-bibliometrix in **Figure 2**. The included publications from 324 journals with an annual publication growth rate of 19.00%. There were 5291 authors, and a single author wrote 15 papers. Authors with international cooperation accounted for 25.63%. Each article had an average of 7-8 authors; 1485 author’s keywords were provided, and 38596 references were cited. The average life span of each paper from being noticed to being unknown was 4.38 years; each paper had been cited an average of 57-58 times. Additionally, these publications contained a total of 45,102 citations, with an average of 57.24 citations per paper. The H-index for all publications was 107.

### 3.2 The annual trend of global publication quantity

**Figure 3:**
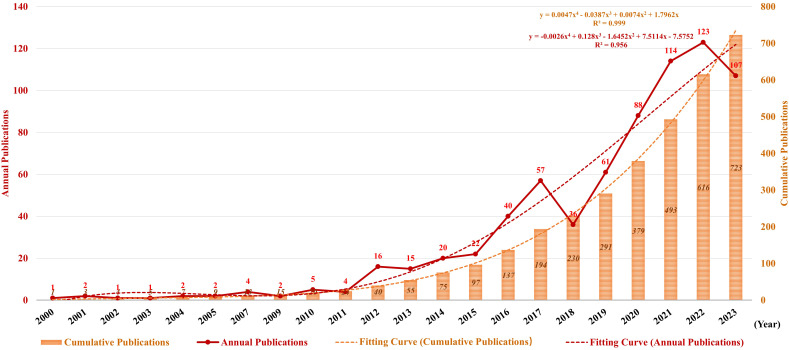
Annual and cumulative growth trend of publications. The red and orange dotted lines represent the trend-fitted curves using polynomial regression model. The correlation coefficients (R^2^) are displayed in the figure.

The change in the number of annual publications characterizes the pace and advancement of research on this topic and the level of attention paid to this area of research. **Figure 3** illustrates the publication trend over the last 20 years, which reveals a circuitous and significant surge in the annual volume of publications in this field after 2015. With the fast increase in the number of annual publications, there were 713 articles on TAN published since 2015, accounting for 90.48% in the past two decades. The publication rate has remained high, with over 50 publications per year since 2019. What’s more, the polynomial curve shows a statistically significant and positive correlation between the number of annual/cumulative publications and the year of publication (R^2^ = 0.999 and R^2^ = 0.956, respectively). According to the fitting curve, the quantity of annual/cumulative publications will be 125 and 890, respectively, in 2024. These findings indicate that TAN is becoming a significant focus of TME research and theories about TAN are booming. Since TAN has gained increasing interest from more scholars in recent years, a spurt will occur shortly.

### 3.3 Country/region and institution analysis

**Figure 4:**
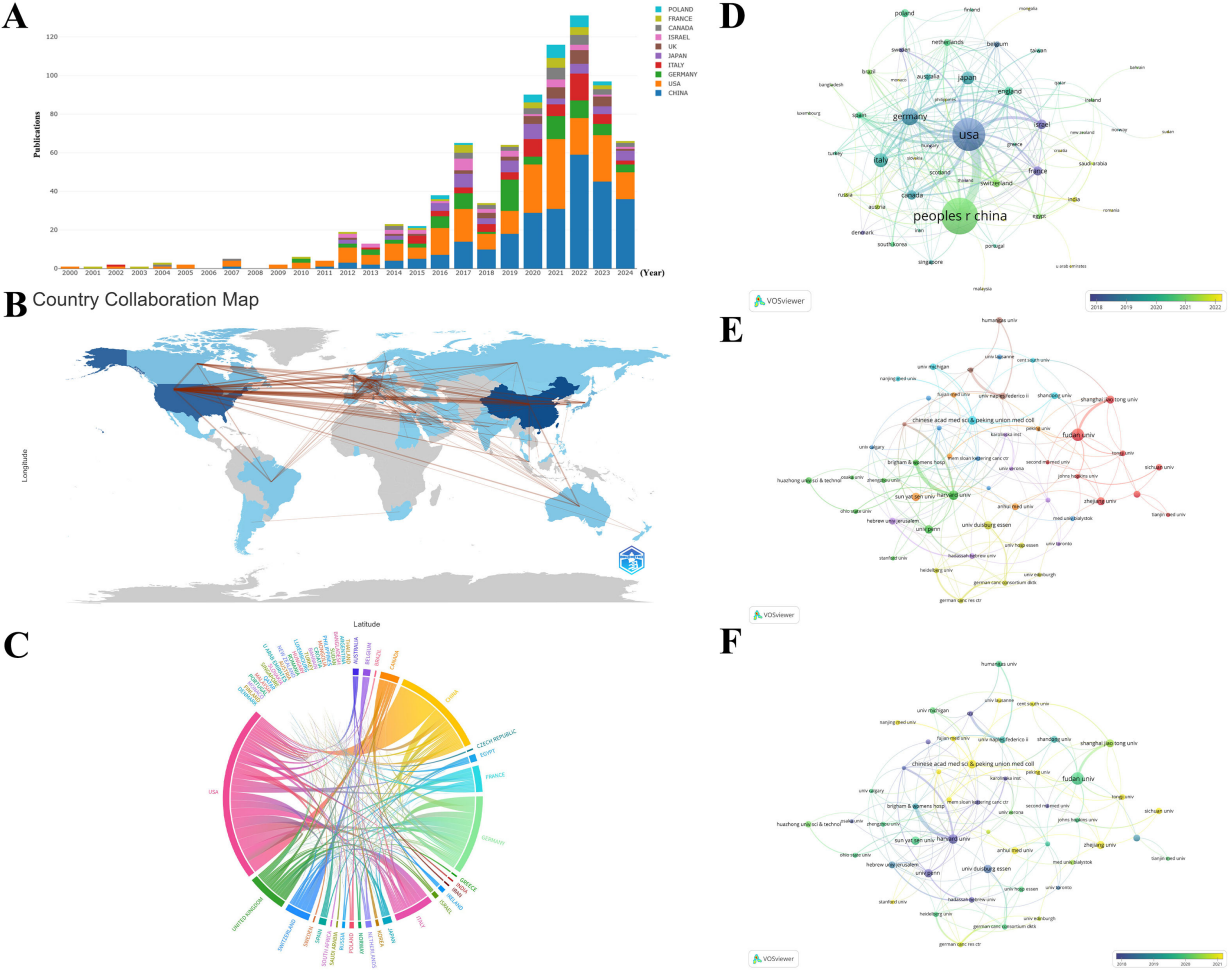
**(A)** The trend of annual number of publications from top 10 countries/regions. **(B)** Geographic distribution of publications across different countries/regions and their international collaborations. The darker blue indicates more publications, and the thicker lines between countries/regions indicate stronger collaborations. **(C)** The international collaborations visualization of countries/regions. The thicker lines between countries/regions indicate stronger collaborations. **(D)** The overlay visualization of country/region co-authorship. The larger nodes represent more publications, and the thicker lines between countries/regions indicate stronger collaborations. The countries/regions in the blue cluster are considered pioneers, whereas the countries/regions in the yellow and green clusters began to publish papers in recent years. **(E)** The co-authorship network among institutions. The divergent colors represent divergent cooperative clusters, with institutions in the same color cluster exhibiting stronger co-authorships. **(F)** The overlay visualization of institution co-authorship. The institutions in the blue cluster are considered pioneers, whereas the institutions in the yellow and green clusters began to publish papers in recent years.

58 countries/regions have published papers on this topic, with China (N=255), the United States (N=213), and Germany (N=77) contributing the most (**Figures 4A, B**; **Table 1**). The interactive cooperation map plots the extensive cooperation relationships between countries/regions (**Figures 4B, C**). The top countries with the highest number of publications, such as China, the United States, Germany, and Italy, have notably close partnerships with other countries/regions, especially the closest relationship established between China and the United States among all countries. Despite producing more papers than the United States, China did not receive higher citations and total link strength (TLS) than the United States, partially because China recently has cut a striking figure as an emerging country in the realm according to **Figure 4D**. The United States is the international cooperation center with the highest TLS (N=151) in this field, but also had the highest citations (N=18496) as shown in **Table 1**.

**Table 1:** The top 10 countries/regions and institutions ranked by publications and citations.

Rank	Country	Publications	TLS	Country	Citations	TLS	Institution	Publications	TLS	Institution	Citations	TLS
1	China	255	60	USA	18496	151	Fudan University	34	58	University of Pennsylvania	5150	63
2	USA	213	151	China	9690	60	Harvard University	22	128	Harvard University	2849	128
3	Germany	77	70	Germany	5271	70	Chinese Academy of Medical Sciences & Peking Union Medical College	19	46	Stanford University	2191	15
4	Italy	56	39	Italy	3671	39	Sun Yat-Sen University	18	26	Hadassah-Hebrew University	1754	11
5	Japan	49	15	Israel	3533	27	University of Duisburg-Essen	17	38	Fudan University	1730	58
6	Canada	30	35	England	2372	48	University of Pennsylvania	17	63	Brigham and Women’s Hospital	1489	58
7	Israel	30	27	Netherlands	1941	25	Shanghai Jiao Tong University	16	23	Netherlands Cancer Institute	1272	42
8	France	29	27	France	1608	27	Zhejiang University	16	20	Humanitas University	1236	23
9	England	26	48	Spain	1451	32	Hebrew University of Jerusalem	13	39	The Wistar Institute	1128	8
10	Poland	23	7	Japan	1426	15	Shandong University	13	25	Ludwig-Maximilians-Universität München	1057	21

TLS, total link strength.

The units with the most publications related to TAN research are listed in **Table 1**. Fudan University held the top rank with the highest number of published papers. Nevertheless, the University of Pennsylvania is the institution with the highest total citations (N=5150). The 50 institutions with at least 6 papers were classified into 8 clusters according to the degree of collaboration (**Figure 4E**). These institutions exhibited close link strengths. The Harvard Medical School (N=128) and University of Pennsylvania (N=63) possess the highest TLS, indicative of active collaboration with other institutions in the field of TAN. However, the TLS of top publication institutions including Chinese Academy of Medical Sciences & Peking Union Medical College, Sun Yat-Sen University, and University of Duisburg-Essen were merely 46, 26, and 38 respectively (**Table 1**), which indicates their relatively weaker connections with other institutions. Wherein Chinese Academy of Medical Sciences & Peking Union Medical College and Sun Yat-Sen University are located in the yellow or chartreuse cluster (**Figure 4F**) and thereby they just initially set foot in the field in recent years, which at least partially explains their low TLS and citations in despite of the top publishments. Hence, it is advised that institutions with the highest publication volumes, such as those mentioned above, should aim to strengthen inter-agency cooperation beyond their current academic circles.

### 3.4 Author and co-cited author analysis

**Figure 5:**
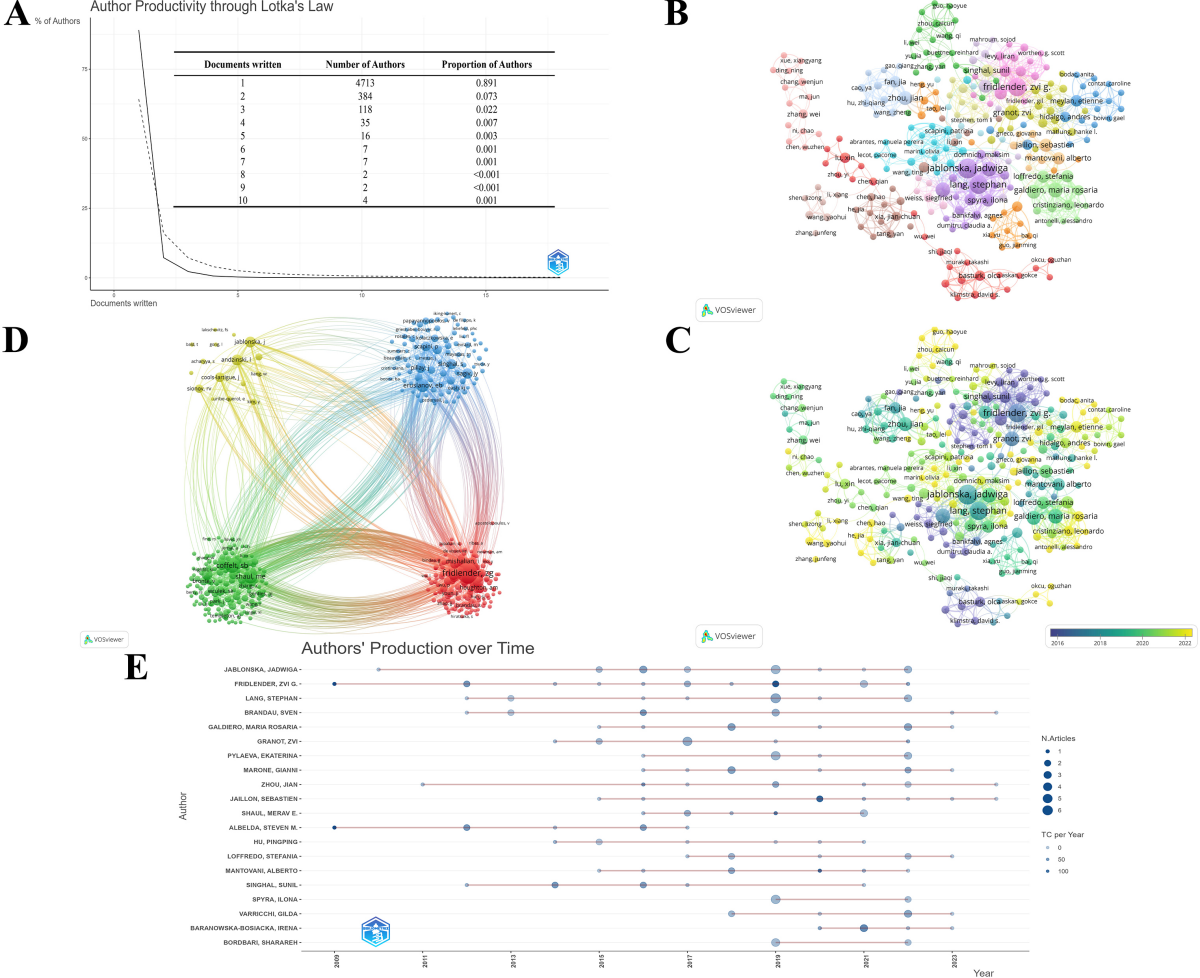
**(A)** Productivity of authors through Lotka’s Law. The solid line represents the actual relationship between the proportion of authors and the quantity of publications they have published, while the dashed line represents the theoretical prediction according to Lotka’s Law. **(B)** The co-authorship network among authors. The larger nodes represent more publications, and the thicker lines between institutions indicate stronger collaborations. The divergent colors represent divergent cooperative clusters, with authors in the same color cluster exhibiting stronger co-authorships. **(C)** The overlay visualization of author co-authorship. The authors in the blue cluster are considered pioneers, whereas the authors in the yellow and green clusters began to publish papers in recent years. **(D)** The co-citation network among authors. The larger nodes denote more co-citations, and the thicker lines denote a stronger co-citation relationship. The authors in same color belong to the same cluster, indicating their work is frequently cited together. **(E)** The world’s publishing career of top 20 TAN researchers. The larger nodes indicate more annual publications, and the darker node shade denotes more annual citations.

There were 5291 authors involved in the study of TAN. Scientific productivity through Lotka’s law shows that 89.1% of authors contributed solely single publication (**Figure 5A**). The 257 authors with co-authorship are depicted in **Figure 5B**. It is conspicuous from the figure that close collaboration and communication among distinct clusters are lacking, denoting again that the researchers in this field should strengthen their interagency and international cooperation efforts. When combined with the overlay visualization map of author co-authorship analysis (**Figure 5C**), it can be observed that the authors in the blue cluster are considered pioneers in the field of TAN, whereas the authors in the red and orange clusters began to publish papers in recent years.

Moreover, a co-citation analysis is performed to illustrate the functional and thematic influence of authors who have been cited more than 20 times in the field of TAN (**Figure 5D**), which generates a network of 401 interconnected authors. Apparently, Fridlender, Zvi G., Mantovani, Alberto, and Coffelt, Seth B. are the top three co-cited authors in this analysis, showing their dominance in this field. Furthermore, it is noteworthy that highly productive authors tend to appear together more than others.

Meanwhile, R-bibliometrix is employed to explore the authors’ local impact, and **Table 2** highlights the top 10 authors in terms of the number of publications and total citations in which the local H-index attempts to evaluate the authors according to published papers in the TAN study. To further analyze the top 20 TAN researchers with the highest output, the number of papers published each year and the total amount of citations per year from 2000 to 2024 are depicted in the time graph (**Figure 5E**), through which the careers of the researchers can be seen directly. It is straightforward to deduce that the scientific research output of Fridlender, Zvi G. has been distributed discontinuously from 2009 to recent years, with a relatively high number of papers published and the number of citations, whereas the most other prolific authors have come to prominence in the past decade.

**Table 2:** The top 10 authors ranked by publications and citations.

Rank	Author	Publications	Citations	H-index	Cited author	Citations	Publications	H-index
1	Jablonska, Jadwiga	18	1026	14	Fridlender, Zvi G.	4775	15	14
2	Fridlender, Zvi G.	15	4775	14	Albelda, Steven M.	3766	7	7
3	Lang, Stephan	15	596	12	Sun, Jing	2718	5	5
4	Brandau, Sven	10	843	10	Kapoor, Veena	2697	3	3
5	Galdiero, Maria Rosaria	10	688	10	Cheng, Guanjun	2534	2	2
6	Granot, Zvi	10	818	10	Worthen, G. Scott	2534	2	2
7	Pylaeva, Ekaterina	10	297	9	Kim, Samuel	2290	1	1
8	Marone, Gianni	9	672	8	Ling, Leona	2290	1	1
9	Zhou, Jian	9	1091	7	Alizadeh, Ash A.	2096	1	1
10	Jaillon, Sebastien	8	892	7	Diehn, Maximilian	2096	1	1

### 3.5 Journal and co-cited journal analysis

**Figure 6:**
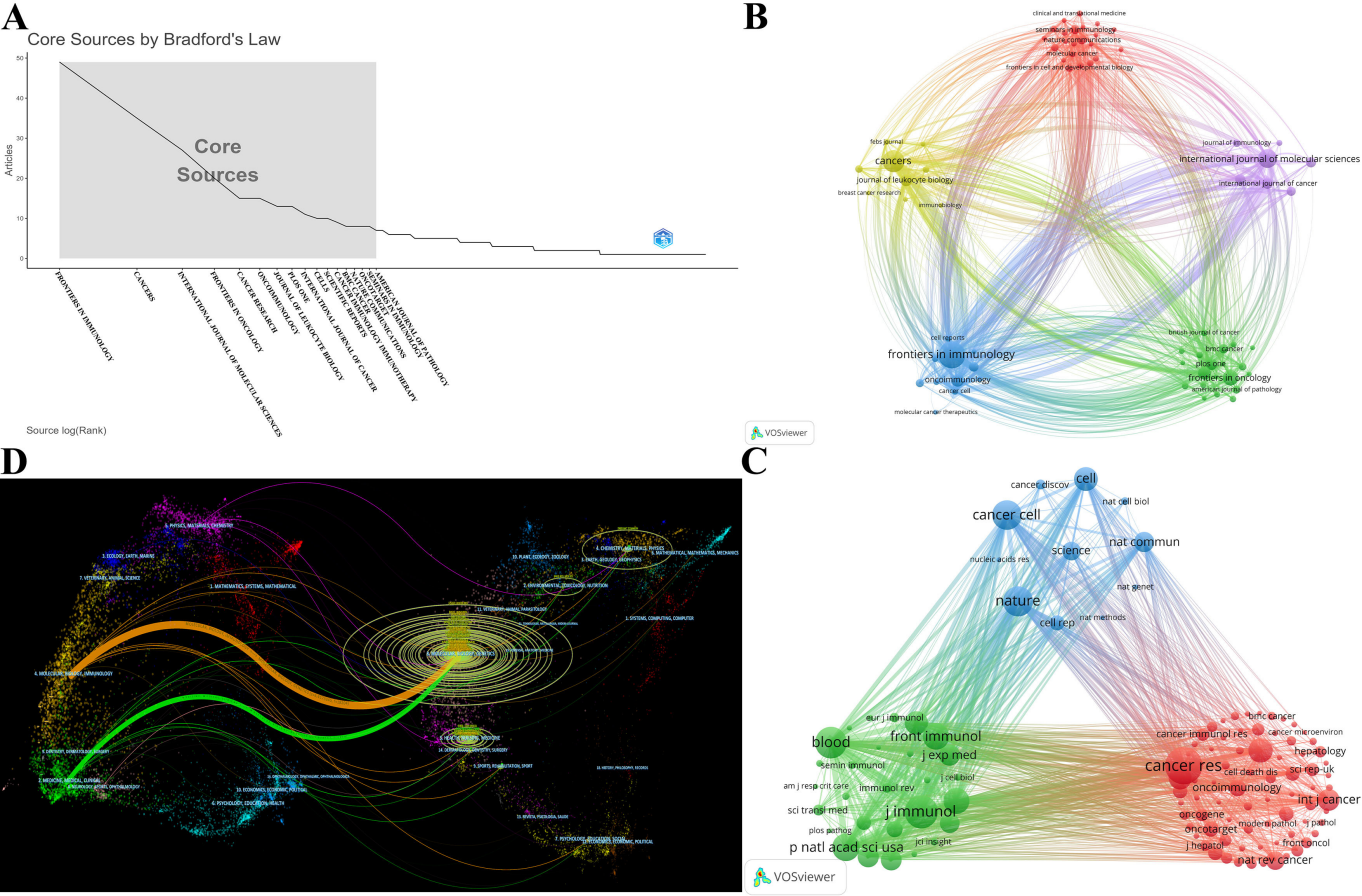
**(A)** 17 core journals based on Bradford’s Law. **(B)** The citing network among journals. The larger nodes denote more publications, and the thicker lines indicate stronger cross-citation relationships between two journals. **(C)** The co-cited network among journals. The larger nodes represent more co-citations. The journals in same color belong to the same cluster, and there is an analogous theme among journals in the same cluster. **(D)** The dual-map overlay of journals. The colored path from left to right depicts the citation pathways. The journals that cite articles are located on the left side, whereas the journals that are cited are on the right side. The labels close to the emitting region represent the respective disciplines, with each label centered around the cluster centroid of the corresponding journals. The longer the vertical axis of the ellipse, the more papers the journal published; and the longer the horizontal axis, the more authors it had.

These publications were sourced from 324 journals. A total of 208 publications were covered in the top 10 prolific journals, representing 26.40% of all publications (**Table 3**). 5 of the top 10 prolific journals have an impact factor (IF) greater than 5, and 6 of the top 10 prolific journals were at the Q1 based on the 2023 Journal Citation Reports (JCR). Notably, 17 journals were identified as core journals using Bradford’s Law (**Figure 6A**), comprising 266 publications. The 69 journals with a minimum of 3 relevant publications created a citing network diagram and formed 5 clusters as shown in **Figure 6B**. Apparently, the journals in which TAN research results are published have active citation relationships. CANCER CELL had the highest citations (N=2950) and the second highest TLS (N=574) despite barely 5 publications, whereas FRONTIERS IN IMMUNOLOGY had the highest number both of publications (N=49) and TLS (N=828) (**Figure 6B**; **Table 3**), demonstrating their significant impacts in TAN field.

**Table 3:** The top 10 most prolific journal and highly co-cited journal.

Rank	Journal	Documents	Citations	IF (2023)/JCR division	Co-cited Journal	Citations	IF (2023)/JCR division
1	FRONTIERS IN IMMUNOLOGY	49	2503	5.7/Q1	CANCER RESEARCH	2522	12.5/Q1
2	CANCERS	35	1034	4.5/Q2	JOURNAL OF IMMUNOLOGY	2075	3.6/Q2
3	INTERNATIONAL JOURNAL OF MOLECULAR SCIENCES	27	1047	4.9/Q1	BLOOD	1965	21.0/Q1
4	FRONTIERS IN ONCOLOGY	20	659	3.5/Q2	NATURE	1817	50.5/Q1
5	CANCER RESEARCH	15	1665	12.5/Q1	CANCER CELL	1736	48.8/Q1
6	ONCOIMMUNOLOGY	15	768	6.5/Q1	PROCEEDINGS OF THE NATIONAL ACADEMY OF SCIENCES OF THE UNITED STATES OF AMERICA	1567	9.4/Q1
7	JOURNAL OF LEUKOCYTE BIOLOGY	13	595	3.6/Q2	FRONTIERS IN IMMUNOLOGY	1463	5.7/Q1
8	PLOS ONE	13	1009	2.9/Q1	JOURNAL OF CLINICAL INVESTIGATION	1449	13.3/Q1
9	INTERNATIONAL JOURNAL OF CANCER	11	995	5.7//Q1	CLINICAL CANCER RESEARCH	1298	10.0/Q1
10	CELLS	10	117	5.1/Q2	CELL	1271	45.5/Q1

IF, impact factor; JCR, Journal Citation Reports.

All top 10 co-cited journals, each cited over 1000 times, with CANCER RESEARCH (N=2522 times) being the most co-cited (**Table 3**). Notably, all top 10 co-cited journals also had fairly high academic rankings, and almost all of them were in Q1 and had an IF of 10 or higher, with the highest being Nature, which had an IF of 50.5. Likewise, the journal co-citation network selecting journals with at least 100 citations illustrates the association between two journals (**Figure 6C**). The included 115 journals were classified into 3 clusters. There is an analogous theme between journals of the same color, especially for the red cluster. CANCER RESEARCH had the highest citations and TLS (citations = 2522, TLS = 357227), followed by JOURNAL OF IMMUNOLOGY (citations = 2075, TLS = 343577) and BLOOD (citations = 1965, TLS = 335179), which all had substantial contributions to TAN publications (**Figure 6C**).

A dual-map overlay of journals portrays the topic distribution of scientific journals and illustrates the primary citation connections between citing and cited journals, with the colored path from left to right depicting the citation pathways. As depicted in **Figure 6D**, the citing journals on the left side of the map are chiefly from Molecular/Biology/Immunology and Medicine/Medical/Clinical, called research frontiers; whereas the cited journals on the right side are chiefly from Molecular/Biology/Genetics, called the knowledge base.

### 3.6 Document citation analysis

**Figure 7:**
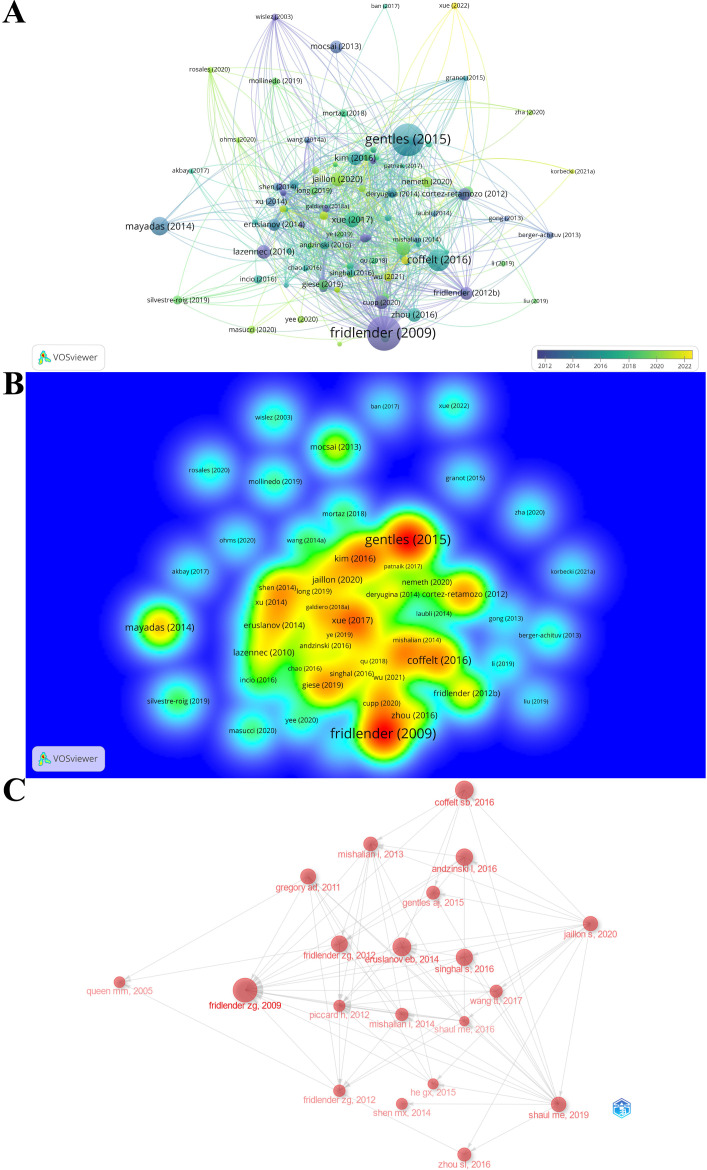
**(A)** The overlay visualization map of document citation analysis. The larger nodes represent more citations, and the thicker lines indicate stronger cross-citation relationships between two documents. The color denotes the publication year. **(B)** The spectral density map of document citation. The redder color represents more citations. **(C)** Association between the top 20 citation bursts. The larger nodes represent more citations, and the arrows denote citing direction.

The citation score of 788 documents was analyzed by the R-bibliometrix, and the top 20 local citation score (LCS) and global citation score (GCS) documents are listed in **Table 4**. Specifically, for a certain literature, LCS is the number of citations in the exported local database, which reflects the impact in this fields, whereas GCS is the total number of citations in the Web of Science database, which reveals the impact in all fields. There are 11 documents simultaneously listed in the top 20 of “Most Local Cited Documents” and “Most Global Cited Documents”. Of them, Fridlender, Zvi G. published the article (Polarization of tumor-associated neutrophil phenotype by TGF-beta: “N1” versus “N2” TAN) with the highest citation frequency both locally and globally (LCS: 396, GCS: 2290) in CANCER CELL in 2009, when the area just emerged. The above results illustrate that this document had a significant impact on the TAN field. The possible reason is that this incipient document concentrated on the basic biological characteristics of TAN within the TME and suggests that at least 2 different polarized populations of TANs are similar to what is seen in macrophages. In this paper, Fridlender, Zvi G. firstly introduced a classification scheme for TAN similar to that of tumor-associated macrophage: TAN can thus take an anti-tumorigenic (what we are calling an “N1-phenotype”) versus a pro-tumorigenic (“N2”) phenotype (15), and this paradigm could explain some of the apparent contradictions in the evaluation of the role of neutrophils in tumor biology, which proffers reference and theoretical basis for subsequent in-depth study in characteristics and biological functions of TAN within the TME. After that, scholars around the world conducted more and more research based on this classical research. What is more, Fridlender, Zvi G.’s next two publications in 2012 also ranked in the top 6 and top 15 most local cited documents, and won the high LCS scores of 133 and 70, respectively. In reality, combined with the world’s publishing career of the top TAN researchers mentioned above, we may safely conclude that Fridlender, Zvi G. is the founder, pioneer, and cultivator of the TAN area and his research outputs have been leading the trend and breakthrough in this area. Likewise, the citation relationship of 112 documents with at least 100 citations is also portrayed in the overlay visualization map (**Figure 7A**), while the impact of documents in the field is demonstrated in the density **Figure 7B**. Specifically, **Figure 7C** maps the relationship of the top 20 publications by local citation frequency.

**Table 4:** Citation score of documents (Top 20).

Rank	Most local cited documents				Most global cited documents	
	Title First author Journal Date	LCS	GCS	LC/GC Ratio (%)	Title First author Journal Date	GCS
1	Polarization of tumor-associated neutrophil phenotype by TGF-beta: “N1” versus “N2” TAN Fridlender, Zvi G. CANCER CELL 2009	396	2290	17.29	Polarization of tumor-associated neutrophil phenotype by TGF-beta: “N1” versus “N2” TAN Fridlender, Zvi G. CANCER CELL 2009	2290
2	Tumor-associated neutrophils stimulate T cell responses in early-stage human lung cancer Eruslanov, Evgeniy B. JOURNAL OF CLINICAL INVESTIGATION 2014	180	439	41.00	The prognostic landscape of genes and infiltrating immune cells across human cancers Gentles, Andrew J. NATURE MEDICINE 2015	2096
3	Neutrophils in cancer: neutral no more Coffelt, Seth B. NATURE REVIEW CANCER 2016	176	1144	15.38	Neutrophils in cancer: neutral no more Coffelt, Seth B. NATURE REVIEW CANCER 2016	1144
4	Type I IFNs induce anti-tumor polarization of tumor associated neutrophils in mice and human Andzinski, Lisa INTERNATIONAL JOURNAL OF CANCER 2016	145	286	50.70	The multifaceted functions of neutrophils Mayadas, Tanya N. ANNUAL REVIEW OF PATHOLOGY-MECHANISMS OF DISEASE 2014	847
5	Origin and role of a subset of tumor-associated neutrophils with antigen-presenting cell features in early-stage human lung cancer Singhal, Sunil CANCER CELL 2016	139	282	49.29	Tumor-associated macrophages and neutrophils in TME Kim, Jaehong MEDIATORS OF INFLAMMATION 2016	562
6	Tumor-associated neutrophils: friend or foe? Fridlender, Zvi G. CARCINOGENESIS 2012	133	498	26.71	Neutrophil diversity and plasticity in tumour progression and therapy Jaillon, Sebastien NATURE REVIEWS CANCER 2020	539
7	Tumor-associated neutrophils: new targets for cancer therapy Gregory, Alyssa D. CANCER RESEARCH 2011	113	532	21.24	Tumor-associated neutrophils recruit macrophages and T-regulatory cells to promote progression of hepatocellular carcinoma and resistance to sorafenib Zhou, Shaolai GASTROENTEROLOGY 2016	534
8	Tumour-associated neutrophils in patients with cancer Shaul, Merav E. NATURE REVIEWS CLINICAL ONCOLOGY 2019	105	531	19.77	Tumor-associated neutrophils: new targets for cancer therapy Gregory, Alyssa D. CANCER RESEARCH 2011	532
9	Neutrophil diversity and plasticity in tumour progression and therapy Jaillon, Sebastien NATURE REVIEWS CANCER 2020	103	539	19.11	Tumour-associated neutrophils in patients with cancer Shaul, Merav E. NATURE REVIEWS CLINICAL ONCOLOGY 2019	531
10	Tumor-associated neutrophils develop pro-tumorigenic properties during tumor progression Mishalian, Inbal CANCER IMMUNOLOGY, IMMUNOTHERAPY 2013	93	247	37.65	Tumor-associated neutrophils: friend or foe? Fridlender, Zvi G. CARCINOGENESIS 2012	498
11	Tumor-associated neutrophils recruit macrophages and T-regulatory cells to promote progression of hepatocellular carcinoma and resistance to sorafenib Zhou, Shaolai GASTROENTEROLOGY 2016	85	534	15.92	Origins of tumor-associated macrophages and neutrophils Cortez-Retamozo, Virna PROCEEDINGS OF THE NATIONAL ACADEMY OF SCIENCES OF THE UNITED STATES OF AMERICA 2012	492
12	The prognostic landscape of genes and infiltrating immune cells across human cancers Gentles, Andrew J. NATURE MEDICINE 2015	83	2096	3.96	Tumor-associated neutrophils stimulate T cell responses in early-stage human lung cancer Eruslanov, Evgeniy B. JOURNAL OF CLINICAL INVESTIGATION 2014	439
13	Neutrophils recruit regulatory T-cells into tumors via secretion of CCL17–a new mechanism of impaired anti-tumor immunity Mishalian, Inbal INTERNATIONAL JOURNAL OF CANCER 2014	77	176	43.75	Neutrophils as emerging therapeutic targets Németh, Tamás NATURE REVIEWS DRUG DISCOVERY 2020	374
14	Tumour-activated neutrophils in gastric cancer foster immune suppression and disease progression through GM-CSF-PD-L1 pathway Wang, Ting-Ting GUT 2017	76	306	24.84	Neutrophil plasticity in the TME Giese, Morgan A. BLOOD 2019	369
15	Transcriptomic analysis comparing tumor-associated neutrophils with granulocytic myeloid-derived suppressor cells and normal neutrophils Fridlender, Zvi G. PLOS ONE 2012	70	244	28.69	Heterogeneity of neutrophils Ng, Lai Guan NATURE REVIEWS IMMUNOLOGY 2019	365
16	On the dual roles and polarized phenotypes of neutrophils in tumor development and progression Piccard, H. CRITICAL REVIEWS IN ONCOLOGY HEMATOLOGY 2012	68	257	26.46	Tumor associated neutrophils. Their role in tumorigenesis, metastasis, prognosis and therapy Masucci, Maria Teresa FRONTIERS OF ONCOLOGY 2019	343
17	Tumor-associated neutrophils as a new prognostic factor in cancer: a systematic review and meta-analysis Shen, Meixiao PLOS ONE 2014	67	228	29.39	Tumour-activated neutrophils in gastric cancer foster immune suppression and disease progression through GM-CSF-PD-L1 pathway Wang, Ting-Ting GUT 2017	306
18	Breast cancer cells stimulate neutrophils to produce oncostatin m: potential implications for tumor progression Queen, Mm CANCER RESEARCH 2005	66	297	22.22	Breast cancer cells stimulate neutrophils to produce oncostatin m: potential implications for tumor progression Queen, Mm CANCER RESEARCH 2005	297
19	Peritumoural neutrophils negatively regulate adaptive immunity via the PD-L1/PD-1 signalling pathway in hepatocellular carcinoma He, Gaixia JOURNAL OF EXPERIMENTAL & CLINICAL CANCER RESEARCH 2015	64	171	37.43	Neutrophils in cancer: two sides of the same coin Uribe-Querol, Eileen JOURNAL OF IMMUNOLOGY RESEARCH 2015	291
20	Tumor-associated neutrophils display a distinct N1 profile following TGFβ modulation: A transcriptomics analysis of pro- vs. anti-tumor TANs Shaul, Merav E. ONCOIMMUNOLOGY 2016	63	163	38.65	Neutrophils in homeostasis, immunity, and cancer Angel Nicolas-Avila, Jose IMMUNITY 2017	291

LCS, local citation score; GCS, global citation score.

### 3.7 Co-cited reference analysis

**Figure 8:**
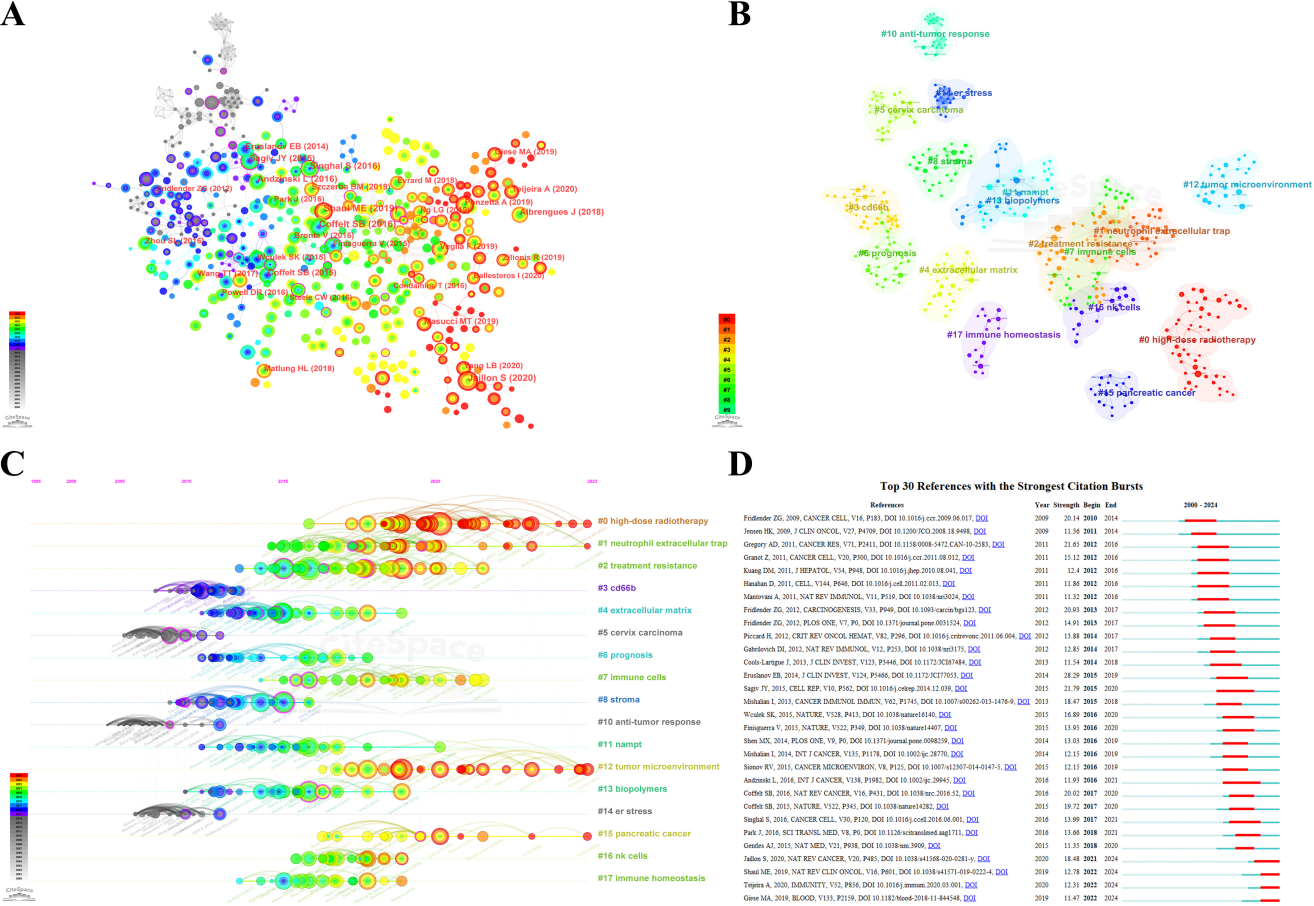
**(A)** Co-citation analysis of the references. The larger nodes and labels are assigned to references with more co-citations, and the thicker lines represent stronger co-citation relationships. The thicker node periphery (purple circle) represents higher intermediate centricity. **(B)** Clustering network analysis of co-cited references. The clusters apply keywords of references as label source. The clusters are numbered in ascending order, and smaller numbers indicating more studies within the corresponding cluster. **(C)** The timeline view of co-cited references analysis. The nodes appear on the timelines in diverse positions and colors, based on the time of reference publication. The nodes on the left represent earlier references, whereas those on the right represent more recent ones. The nodes positions along the same line represent a cluster, identified by the label # on the right side; the length of the horizontal line and fore and aft ends are the duration for that category. **(D)** Top 30 references with the strongest citation bursts from 2000 to 2024. The green lines represent the period from 2000 to 2024, whereas the red lines demonstrate the duration of each citation burst during which the references were cited most.

The reference co-citation network is depicted in **Figure 8A**, where a scaling factor k=15 was set and 676 cited literature references were extracted using g-index. The labels showed the first author with the thirty most cited references and the year. Additionally, considering the 10 most co-cited publications were also of interest to most TAN researchers, they are listed in **Table 5**.

**Table 5:** The top 10 co-cited references involved in the field of TAN.

Rank	Co-cited reference	Citations	Centrality	First author	Year	Type	Journal	IF(2023)/JCR division
1	Neutrophils in cancer: neutral no more	103	0	Coffelt, Seth B.	2016	Review	NATURE REVIEWS CANCER	72.5/Q1
2	Tumor-associated neutrophils in patients with cancer	102	0	Shaul, Merav E.	2019	Review	NATURE REVIEWS CLINICAL ONCOLOGY	81.1/Q1
3	Neutrophil diversity and plasticity in tumour progression and therapy	101	0	Jaillon, Sebastien	2020	Review	NATURE REVIEWS CANCER	72.5/Q1
4	Type I IFNs induce anti-tumor polarization of tumor associated neutrophils in mice and human	88	0.08	Andzinski, Lisa	2016	Article	INTERNATIONAL JOURNAL OF CANCER	5.7/Q1
5	Origin and role of a subset of tumor-associated neutrophils with antigen-presenting cell features in early-stage human lung cancer	81	0.17	Singhal, Sunil	2016	Article	CANCER CELL	48.8/Q1
6	Neutrophil extracellular traps produced during inflammation awaken dormant cancer cells in mice	77	0	Albrengues, Jean	2018	Article	SCIENCE	44.7/Q1
7	Phenotypic diversity and plasticity in circulating neutrophil subpopulations in cancer	73	0.23	Sagiv, Jitka Y.	2015	Article	CELL REPORTS	7.5/Q1
8	Tumor-associated neutrophils stimulate T cell responses in early-stage human lung cancer	69	0	Eruslanov, Evgeniy B.	2014	Article	JOURNAL OF CLINICAL INVESTIGATION	13.3/Q1
9	CXCR1 and CXCR2 chemokine receptor agonists produced by tumors induce neutrophil extracellular traps that interfere with immune cytotoxicity	67	0	Teijeira, Alvaro	2020,	Article	IMMUNITY	25.5/Q1
10	IL-17-producing γδ T cells and neutrophils conspire to promote breast cancer metastasis	62	0.08	Coffelt, Seth B.	2015	Article	NATURE	50.5/Q1

IF, impact factor; JCR, Journal Citation Reports.

The co-citation analysis forms the 17 clusters, and the modularity Q and the mean silhouette S were higher than 0.85, showing a significant clustering effect and a highly credible network (**Figure 8B**). The timeline view illustrates how research hotspots have evolved over time (**Figure 8C**). Overall, the rise in citations in this field and most of the highly cited literature emerged began in 2010, and many of the co-cited references continue to be widely cited, denoting the TAN area remains a prominent research topic that has been making a rapid advancement and a vast array of results. It can be observed that clusters #3 CD66b, #5 cervix carcinoma, #10 anti-tumor response, and #14 er stress are the initial topics of study in this field, but the hotspot has already shifted from them. Whereas the clusters #0 high-dose radiotherapy, #1 neutrophil extracellular trap (NET), #12 tumor microenvironment, and #15 pancreatic cancer are located at the line’s rightmost end, representing the current new hotspots in TAN research.

Herein, reference citation bursts are applied to spotlight the popularity and significance over time of specific references in the TAN field, and the top 30 references with the strongest citation bursts sorted by the beginning year of burst are depicted in **Figure 8D**. We can summarize that co-cited references with high citation bursts predominantly centered on literature with a high IF in which there are 11 reviews in total. The onset of the reference citation burst can be traced back to 2010, attributed to the paper authored by Fridlender, Zvi G., and the latest took place in 2022, with the longest burst spanning 5 years. The article entitled Eruslanov, Evgeniy B., 2014, J CLIN INVEST, V124, P5466 experienced the strongest burst (Strength=28.29). The nature and function of TANs within the TME are largely unclear, and Eruslanov, Evgeniy B. (2014) demonstrated that TANs are not immunosuppressive, but rather stimulate T cell responses in the earliest stages of lung cancer (16). Notably, wherein 3 references with the strongest citation bursts are from Fridlender, Zvi G., which again demonstrates he has made an eminent contribution to the TAN field and laid a solid foundation for subsequent trials. Meanwhile, the most recent burst is attributed to Shaul, Merav E. (2019), Giese, Morgan A. (2019), Jaillon, Sebastien (2020), and Teijeira, Alvaro (2020), which are still undergoing a citation explosion phase even to this day and have the current emergence of strong citation references. Wherein the reviews including Shaul, Merav E. (2019), Giese, Morgan A. (2019), and Jaillon, Sebastien (2020) were respectively published in the NATURE REVIEWS CLINICAL ONCOLOGY, *BLOOD*, and NATURE REVIEWS CANCER, which offer systematic summaries of the significant findings and concepts that have contributed to the field of TAN biology.

### 3.8 Keyword and trend topic analysis

**Figure 9:**
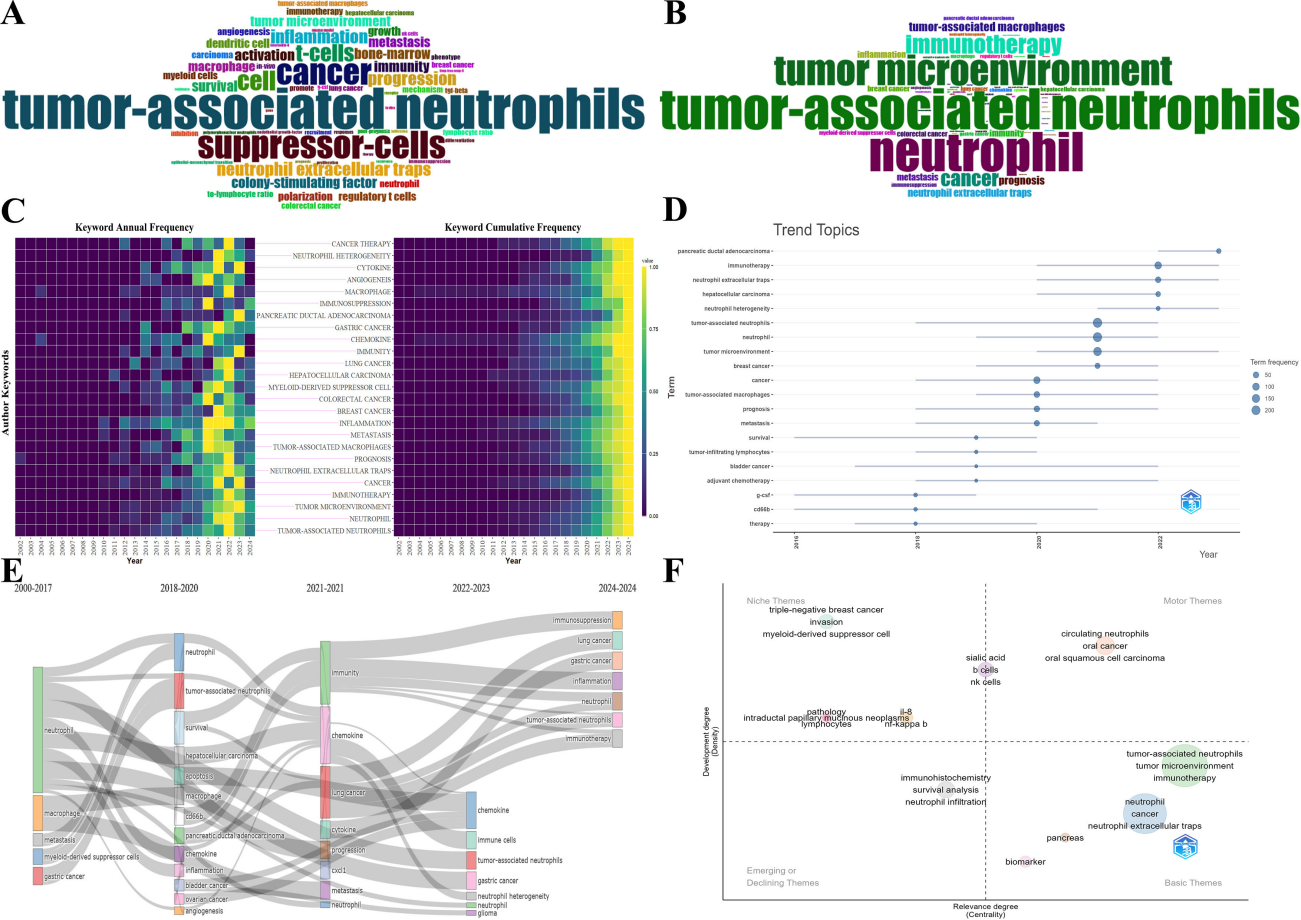
**(A)** The word cloud of top 60 Keywords Plus. The size of words positively correlates with their frequency of occurrence. **(B)** The word cloud of top 60 Author Keywords. The size of words positively correlates with their frequency of occurrence. **(C)** Heatmaps of the top 25 most frequent Author Keywords. Annual keyword frequency on the left and cumulative keyword frequency on the right. **(D)** Keyword-based research theme trend map. It presents each topic as a line whose span represents the duration, with the nodes denoting the most prevalent year for that particular topic. The larger nodes indicate the themes appear more frequently. **(E)** The alluvial diagram of thematic evolution. It unveils the changes and internal links of keywords in different periods. **(F)** The strategic diagram of sub-period. It displays the development trend and maturity level of keywords.

**Figures 9A, B** depicts the word cloud of the top 60 Keywords Plus and Author Keywords, where the size of words shows their frequency of occurrence. The keyword “tumor-associated neutrophil” is in the center of **Figure 9A**, followed by “suppressor-cells,” “cancer,” and so on. Likewise, the results in **Figure 9B** show that “tumor-associated neutrophil” is the most prominent, and “neutrophil” and “tumor microenvironment” are close behind. Obviously, Keywords Plus is broader than Author Keywords. The heatmaps visualize the normalized frequency of Author Keywords from 2000-2024, wherein the annual frequency is on the left and the cumulative frequency is on the right (**Figure 9C**). Additionally, the top 30 most frequent All Keywords are presented in **Table 6**, which generally bespeaks the current focal research.

**Table 6:** Top 30 most frequent All Keywords.

Rank	Keyword	Occurrences	TLS
1	tumor-associated neutrophils	440	5337
2	neutrophil	258	3208
3	cancer	237	2855
4	tumor microenvironment	218	2665
5	suppressor-cells	181	2292
6	expression	144	1683
7	cell	127	1375
8	inflammation	122	1509
9	t-cells	119	1478
10	immunotherapy	114	1442
11	neutrophil extracellular traps	112	1375
12	metastasis	104	1278
13	immunity	103	1260
14	progression	97	1103
15	macrophage	85	992
16	survival	76	839
17	activation	75	887
18	tumor-associated macrophages	73	1028
19	colony-stimulating factor	72	927
20	bone-marrow	71	888
21	breast cancer	69	898
22	prognosis	66	703
23	colorectal cancer	64	754
24	polarization	64	776
25	angiogenesis	63	804
26	dendritic cell	61	816
27	myeloid cells	61	772
28	regulatory t cells	61	820
29	lung cancer	59	724
30	growth	58	635

TLS, total link strength.

The R-bibliometrix visualizes the primary keywords over the past 20 years, analyzing comprehensively the research trends and hotspots. In **Figure 9D**, the research hotspots were relatively homogeneous before 2018, principally focusing on “survival,” “G-CSF,” and “CD66b”. Nevertheless, since 2018, the research hotspots manifested a sudden surge. Specifically, “pancreatic ductal adenocarcinoma,” “immunotherapy,” “neutrophil extracellular traps,” “neutrophil heterogeneity,” and “tumor microenvironment” continue to be the subject of intense research interest. What is more, the emergence of some new keywords in the top-left corner of **Figure 9D**, such as “immunotherapy,” “neutrophil extracellular traps,” “neutrophil heterogeneity,” etc., have gained prominence over the last 2 years, reflecting a discernible shift in the emphasis and direction of future research endeavors. In **Figure 9E**, the alluvial diagram manifests the evolution and internal links of keywords in divergent periods. Apparently, some new topics including “immune cells,” “neutrophil heterogeneity,” “immunosuppression,” “immunotherapy,” etc., have evolved into striking emergence in the past two years, which are basically consistent with the results of the above keyword-based research theme trend map. In **Figure 9F**, the strategic diagram of sub-period presents the development trend and maturity level of keywords. The clusters including “circulating neutrophil,” “oral cancer,” “oral squamous cell carcinoma,” etc., are located in the Motor Themes quadrant, denoting that the above keywords are the core themes with high maturity. Whereas the clusters including “invasion,” “myeloid-derived suppressor cell,” “IL-8,” “NF-kappa-B,” etc., are located in the Niche Themes quadrant, indicating the above keywords are well-developed but banal. Besides, “immunohistochemistry,” “survival analysis,” and “neutrophil infiltration” are located in the Emerging or Declining Themes quadrant, suggesting the above keywords are peripheral themes and have not developed well. On the flip side, the clusters including “tumor microenvironment,” “immunotherapy,” “NETs,” “pancreas,” “biomarker,” etc., are located in the Basic Themes quadrant, demonstrating that the above keywords are significant but the current research is not enough, therefore the above topics may become research hotspots or future development trends.

It is noteworthy that these findings generally align with the outcomes of co-cited reference analysis. Collectively, these findings identified research hotspots and illuminated trends in TAN, revealing a pronounced emphasis on molecular mechanisms in contemporary research.

## 4 Discussion

More than 5 scientific tools were exploited to render a bibliometric map and visualization of 788 TAN-related publications extracted from WoSCC from 2000 to June 1, 2024. Our study systematically appraises the research status and promising future research interests in the field of TAN. To the best of our knowledge, this is the first bibliometric review, summary, and outlook of this field. We exerted quantitative, qualitative, and integrative assessments to identify trends and evolutions in research, which will be canvassed in detail in what follows, delineating a comprehensive overview of our findings.

### 4.1 General information study

The annual publication output wavily ascended over the years without a discernible upward trend until 2015, but there was a significant growth trend with an annual output exceeding 50 papers after 2019. Overall, the field of TAN has made great progress in the past 20 years, thanks to the progress of science and technology studies (especially single-cell multi-omic profiling) and the powerful support from the government. On another level, the yearly escalation in TAN publications is occasioned by the eminent contributions of countries/regions, institutions, authors, and journals.

In terms of geographical distribution, there are merely 58 countries/regions included in our study, and note that less than one-fifth of the countries/regions published more than 20 papers. Moreover, the distribution of institutions is in accordance with countries/regions based on geographical location, while the publication counts for most countries/regions and institutions are out of proportion to their citation counts in this field. Notably, China has emerged as the leading global producer of publications in the field, transcending all other countries/regions, and 6 out of the top 10 active institutions are also located in China. Intriguingly, the United States continues to dominate the field as a global cooperation center with overwhelming citation counts, which evinces the United States had conducted in-depth research in this field and the potentially greater leverage in their articles. As for cooperation between countries, most communication and collaboration were confined to Europe, North America, and a handful of Asian countries.

When it comes to authorship, Israeli professor Fridlender, Zvi G. from the Institute of Pulmonary Medicine, Hadassah-Hebrew University Medical Center is the founder, pioneer, and cultivator of the TAN area with the highest citation counts and local H-index and a significant presence of high LCS papers, therefore his research outputs have been leading the trend and breakthrough and offering reliable reference value for scholars in this field. Our analysis also discloses a disparity between productivity and impact, with some highly productive authors not having a high impact and vice versa. Specifically, just Fridlender, Zvi G. is concurrently in both the top 10 most prolific authors and top 10 most cited authors lists. Notably, Albelda, Steven M. had a high citation counts and local H-index but did not publish a great number of papers, reflecting that he sustains stringent quality control over his publications.

Regarding academic journals, FRONTIERS IN IMMUNOLOGY published the most papers about TAN, implying more meaningful findings are more likely to be published in this journal. In addition, CANCER CELL, FRONTIERS IN IMMUNOLOGY, and NATURE MEDICINE were cited more than 2000 times, with CANCER CELL possessing the most citations, which is indicative of the higher quality as well as better reference value of this journal in this area. Except for JOURNAL OF IMMUNOLOGY, all of the co-cited journals in **Table 3** are located in Q1, demonstrating the significance of TAN in future research.

### 4.2 Emerging trends, hotspots, and frontiers

The performance of reference co-citation networks and keyword clustering outlines underlying research structure in the study of TAN. Through scrupulous examination of the above analyses, a wealth of beneficial insights can be gleaned, encompassing but not confined to TAN heterogeneity, NETs, the interactions between TANs and immunocytes, immunotherapy, and so on. These discoveries allow for the identification of emerging trends and research hotspots within the field of TAN. In subsequent sections, we will canvass their far-reaching meaning for this field of research and latent implications for future orientations.

#### 4.2.1 TAN heterogeneity

Neutrophils consecutively differentiate from the bone marrow into divergent subpopulations, each possessing divergent functions. What is more, in the context of TME, TAN subpopulations display more multifaceted phenotypes and capacities, encompassing angiogenesis, extracellular matrix remodeling, metastasis, and immunomodulation (17–19). The research focuses on TANs have evolved from the macroscopic quantity and function to the microscopic genes and phenotypes. The categorization and detection of divergent phenotypes of TAN symbolize pivotal research hotspots in the field of TAN (20). It is worth noting that the conception of N1 and N2 subpopulations originally introduced by Fridlender, Zvi G. in 2009 to differentiate between anti-tumorigenic and pro-tumorigenic TANs is an oversimplification of their dual phenotypes (15). The latest investigations demonstrated that the simplistic dichotomy of tumor-infiltrating immune cells lacks the requisite specificity and precision to fully describe the heterogeneity of TANs and may not comprehensively depict the whole landscape (21, 22). To date, assorted subpopulations of TAN have been identified in tumors, evincing discrepancies in surface markers as well as functions (23–25). Accordingly, it is imperative to acknowledge the heterogeneity and plasticity of TANs during distinct phases and fathom their anomalous functions within the TME. Although TANs can be perceived as a consequence of neutrophil development and ensuing infiltration into the TME, the prevailing perception of subpopulation categorization remains fragmentary, and it remains hazy how these subpopulations arise from differentiation in the bone marrow, maturation in circulation, or reprogramming within the TME (26). Consequently, implementing a precise subpopulation analysis of TANs has emerged as a compelling focal point warranting further exploration. This ultimately hinges on novel technologies (e.g., genomics, single-cell, and spatial omics) for advancement, which are closely associated with the description of heterogenous transcriptional signatures and potential functional plasticity in TANs. Accurately delineating the provenance of TAN subpopulations and capturing their entire lifespan could furnish scholars with a holistic perception of TAN heterogeneity, which in turn optimizes the timing for interventions in TAN-targeted immunotherapy culminating in an improved patient prognosis. Moreover, a generally recognized separation and identification method for each TAN subpopulation, alongside further perspectives into their functions, is imperative (26). With well-defined notions, isolation procedures, and purification technologies for each TAN subpopulation, patients may be one step closer to the auspicious realm of TAN immunotherapy, akin to the triumph witnessed with CAR-T cell therapy (27–30).

#### 4.2.2 NETs

NETs released by neutrophils consist of decondensed chromatin DNA filaments coated with granule proteins responsible for trapping and killing extracellular pathogens with microbicidal bioactivity via binding to their DNA structures (31, 32), consequently playing a protective role in the antimicrobial defense. Recently, there is growing evidence, however, that NETs also can be induced by tumors through the secretion of varied tumor- and infection-derived molecules, encompassing the overexpression of granulocyte colony-stimulating factor (G-CSF) generally observed in cancer and nicotinamide phosphoribosyl transferase (NAMPT), thereby playing pro-tumorigenic roles in the scenario of cancer-associated inflammation in the majority but not all conditions (33, 34). Wherein NETs induced by G-CSF manifest diversified biomedical behaviors such as capture of circulating tumor cells, epithelial-to-mesenchymal transition, and increment of vascular permeability, which promote cancer-associated thrombosis, tumor intravasation, and metastasis, as well as laminin remodeling and immunosuppressive behaviors, which conduce to tumor proliferation and metastasis (35). On the other hand, NAMPT can stimulate NET formation derived from aged TANs expressing high chemokine receptors 4 and low CD62L, thereby facilitating tumor metastasis (36).

The underlying mechanism of NETs in tumor growth, metastasis formation, and cancer-associated angiogenesis are currently being intensively investigated in full swing. The DNA ingredients of NETs (NET-DNA) in the liver are chemotactic for tumor cells through interplay with high affinity with the coiled-coil domain containing protein 25 (CCDC25) that is a transmembrane protein expressed on tumor cells so that DNA-CCDC25 interplay triggers an intracellular signaling cascade facilitating tumor cell directional migration and metastasis formation (37). Additionally, inflammation facilitates the transition between dormancy and awakening of tumor cells. Specifically, dormant tumor cells adhere to the extracellular matrix protein laminin in murine models with breast and prostate cancers. Repetitive instillation of two NET inducers, including lipopolysaccharide or nicotine (38), bolsters neutrophil inflammation as well as NET formation in the lung. Two NET-related proteases, including neutrophil elastase and matrix metalloproteinase-9, successively cleave laminin exposing an epitope that actuates dormant tumor cell proliferation via integrin activation (38). Furthermore, NETs wrap and coat tumor cells and shield them from the cytotoxicity mediated by CD8^+^ T cells and natural killer (NK) cells (39). Notably, NET-DNA binds transmembrane and coiled-coil domains 6 on CD8^+^ T cells to impede antineoplastic immunity, thereby boosting progression of hepatocellular carcinoma (40). Moreover, as a potential therapeutic target for angiogenesis, angiopoietin induces NETs and thereby elevates tube length and loop quantity in human vascular endothelial cells (HUVECs) (41). The NET-DNA receptor CCDC25 is expressed in HUVECs, proffering a platform for NETs to accelerate HUVEC proliferation, migration, and tubulation (42). NAMPT/SIRT pathway also contributes a lot to NET-associated tumor angiogenesis (43), in which SIRT3 is capable of regulating endothelial cells, thereby stimulating the ROS generation and modulating the hypoxia-inducible factor, culminating in the preferment of angiogenesis (44).

While it is unambiguous that NETs contribute to tumorigenesis, several researchers have begun highlighting also the anti-tumorigenic role of NETs. There is some experimental evidence that NETs suppress the proliferation of colon cancer cells and exert cytotoxic effects on melanoma cells (45, 46). Additionally, NETs forecast elevated survival in patients with head and neck squamous cell carcinoma (47). Whether variations in the quality and quantity of NETs play pro- or anti-tumorigenic roles in divergent types and divergent phases of tumors requires further exploration.

#### 4.2.3 The interactions between TANs and immunocytes

TAN is an integral part of the TME, and its high diversity, heterogeneity and plasticity allow TANs to perform their dual function in tumor immunity via forming an intricate crosstalk with immunocytes. Wherein the crosstalk between TAN and T cell was initially disclosed within the TME. For instance, a distinctive subpopulation of N1 TAN, induced by granulocyte-macrophage colony stimulating factor (GM-CSF) and interferon-γ (IFN-γ) and characterized by CD86 and HLA-DR expression, was identified as possessing antigen-presentation facilities that augment the antineoplastic adaptive immunity of both CD8^+^ T cells and CD4^+^ T cells through MHC-TCR binding (48). Another specific subpopulation of TANs discerned in sarcoma and expressing augmented CD11b and CD54, along with diminished CD62L, has been shown an exceptional cytokine secretion profile, encompassing C-X-C Motif Chemokine Ligand (CXCL)10, interleukin (IL)-23a, and arginase-1, which in conjunction with IL-12 secreted by macrophages synergistically robustly boost the CD4^−^CD8^−^ unconventional αβ T cell polarization and their IFN-γ secretion, thereby triggering a type I immune response against tumor (49). By contrast, there is also literature indicating that TANs can repress the antineoplastic function of T cells and facilitate tumor metastasis (50). Moreover, another subpopulation of programmed cell death 1 ligand^+^ (PD-L1^+^) TANs, differentiated by lactate, can curb T cell cytotoxicity against tumors (51, 52). Given the dual regulatory effect on T cells by TANs, it is imperative to exactly differentiate the specific subpopulations for the exploitation of immunotherapies that complement T-cell anti-tumor immunity.

TANs can also communicate with tumor-infiltrating B cells in varied cancers, resulting in consequential transitions in the behavior of plasma cells (53). TANs secrete the cytokine B-cell-activating factor (BAFF) and a proliferation-inducing ligand (APRIL), which conduce to B cell recruitment but also the IgM generation, along with its switching to IgG or IgA (54). Noticeably, except for molecules encompassing cytokine BAFF, APRIL, and IL-21, TNF-α secreted by TANs also plays a role in inducing B cell chemotaxis, which bolsters the movement and migration of B cells along with CXCL12 or CXCL13 (31, 55). Furthermore, TANs are implicated in the neoplastic B cell differentiation, which also can be mediated by the aforementioned pertinent molecules, like APRIL. Given the manifold effects of B cells in anti-tumor immunity, encompassing direct antineoplastic function via antibody-dependent cellular cytotoxicity and indirect function via activating other immune cells (e.g., T cells and NK cells) (56, 57), it is imperative to investigate whether and how TANs participate in these interactions. Simultaneously, it introduces an intriguing problem pertaining to whether TANs are related to the immunosuppressive function of B cells, encompassing tumor angiogenesis, lymphotoxin generation, and T cell suppression (58–60).

The convoluted interplays between TANs and other innate cells mostly are presumed to exert immunosuppressive effects, while the latent mechanism remains substantially unexplored and warrants further investigations. Fascinatingly, TANs repress tumor metastasis without NK cell, while the crosstalk between TANs and NK cells may facilitate breast cancer metastasis (61). Moreover, the dynamic interaction between TANs and tumor-associated macrophages within the TME collaboratively advances the progression of intrahepatic cholangiocarcinoma via activating signal transducer and activator of transcription 3 (62). Another subpopulation of CCL3^+^CCL4^+^ TAN induced by GM-CSF and IL-6 recruits macrophages by secreting chemokines and thereby promoting tumor metastasis, albeit the ambiguous phenotypic characteristics of the above macrophages (51).

#### 4.2.4 Immunotherapy

The key implication of TANs in the immunotherapy realm has been preliminarily expounded via several retrospective cohort studies. Patients afflicted with hepatocellular carcinoma were observed a high expression of programmed cell death protein 1 (PD-1) in both intratumoral and peritumoral TANs, exceeding that detected in circulating neutrophil populations (63). These particular TANs repressed T cell proliferation and activation, thereby accentuating their necessity in the case of PD-1-based immunotherapies. Nevertheless, fascinatingly, the intratumoral TAN counts in melanoma patients treated with the anti-PD-1 antibody, nivolumab, did not significantly deviate from those of non-users (64). Additionally, the administration of nivolumab was correlated with escalated infiltration density of TANs in patients afflicted with pancreatic ductal adenocarcinoma, which pertains to worse overall survival (65). The latest scientific endeavors have proffered a more nuanced insight, positing that the hitherto conventional beliefs may have been somewhat unilateral and that certain particular TAN subpopulations exert a conspicuous imprint on immunotherapy efficacy. For instance, the efficacious immunotherapy provoked a fleeting upsurge in the TAN abundance, which outlined a characteristic cohort of therapy-induced TANs expressing dominantly an interferon gene signature and is a peculiarity regarded integral for the immunotherapeutic regimens (66). Furthermore, albeit the deficiency of target antigen Trp1 in the bulk of melanoma cells, T-cell therapy efficaciously annihilated these immune-escaped melanoma cells with the aid of a subpopulation of TANs possessing the capacity to secrete nitric oxide in a murine model (64). The exact ramifications of TANs in immunotherapies are still contentious. Nevertheless, we can presume that the immunotherapeutic efficacy may be remarkably impacted by particular TAN subpopulations. The precise mechanisms underlying the interplay between immunotherapy and TANs within the TME are yet to be allsidedly studied. Collectively, TAN is an attractive and fresh immunotherapeutic target (12, 67–70), and a precise delineation as well as accurate targeting of TAN subpopulations doubtlessly hold promise as vital parameters of optimized current immunotherapeutic efficacy. Perhaps the eventual remaining destination in the realm is to translate TAN-targeted immunotherapies into clinical practice.

### 4.3 Limitations

This study inevitably is not without its limitations. First, the search was limited to publications in the core dataset of the Web of Science database, which means certain pertinent papers in other data sources (PubMed, Embase, Scopus, etc.) possibly have been excluded. Secondly, the focus was only on English-language literature so that high-quality articles published in other languages may have been omitted, introducing selection bias. Thirdly, the recently published high-quality literature with a low citation rate may not fully encapsulate its academic merit and not appear in our analysis due to the temporal restriction of our analysis. Lastly, the bibliometric techniques utilized here are limited to metadata rather than full-text analysis so that we may have missed critical insights only present in the full articles, such as author perspectives and outlook on the field. Although the above constraints would not change the results of this study, future work should expand the research base to include non-English studies and the latest outstanding publications.

## 5 Conclusions

A comprehensive bibliometric and visual analysis is first performed to map the current landscape and intellectual structure of TAN. Based on the systematical analysis, it is irrefutable that TANs play a significant role in the TME. The number of publications about TANs has been significantly surging in the past 20 years, denoting that research on TAN is experiencing a vibrant and rapidly evolving stage. Despite China’s maximum quantity of publications and top 10 institutions, the United States is the leading country with the highest impact and is also the global cooperation center. Israeli professor Fridlender, Zvi G. is the founder, pioneer, and cultivator in the TAN area. Our analysis prefigures the future trajectories: TAN heterogeneity, NETs, the crosstalk between TANs and immunocytes, and immunotherapy will likely be the focal points of future research in the field of TAN. Most of all, wherein the TAN heterogeneity in the context of tumor-related pathology exerts a pivotal influence on NETs, the crosstalk between TANs and immunocytes, and immunotherapy, therefore divergent TAN subpopulations could have completely divergent NETs, intercellular crosstalk, and immunotherapeutic implication. Hence, future endeavors must precisely delineate the origin of TAN subpopulations and comprehend their complete lifespan. The accurate identification of distinct TAN subpopulations and the precise targeting of key pro-tumor/anti-tumor subpopulations hold immense potential to develop into a TAN-targeted immunotherapy.

## Data Availability

The original contributions presented in the study are included in the article/supplementary material. Further inquiries can be directed to the corresponding author.
